# Supplementary material for: A streamlined and comprehensive protocol for the generation and multi-omic analysis of human monocyte-derived macrophages
Source: BMC Biotechnol. 2025 Dec 29;25:141. doi: 10.1186/s12896-025-01071-4 (PMC12752039; doi:10.1186/s12896-025-01071-4)
Supplement: Supplementary file 8 — Supplementary Material 8 [file 12896_2025_1071_MOESM8_ESM.docx]

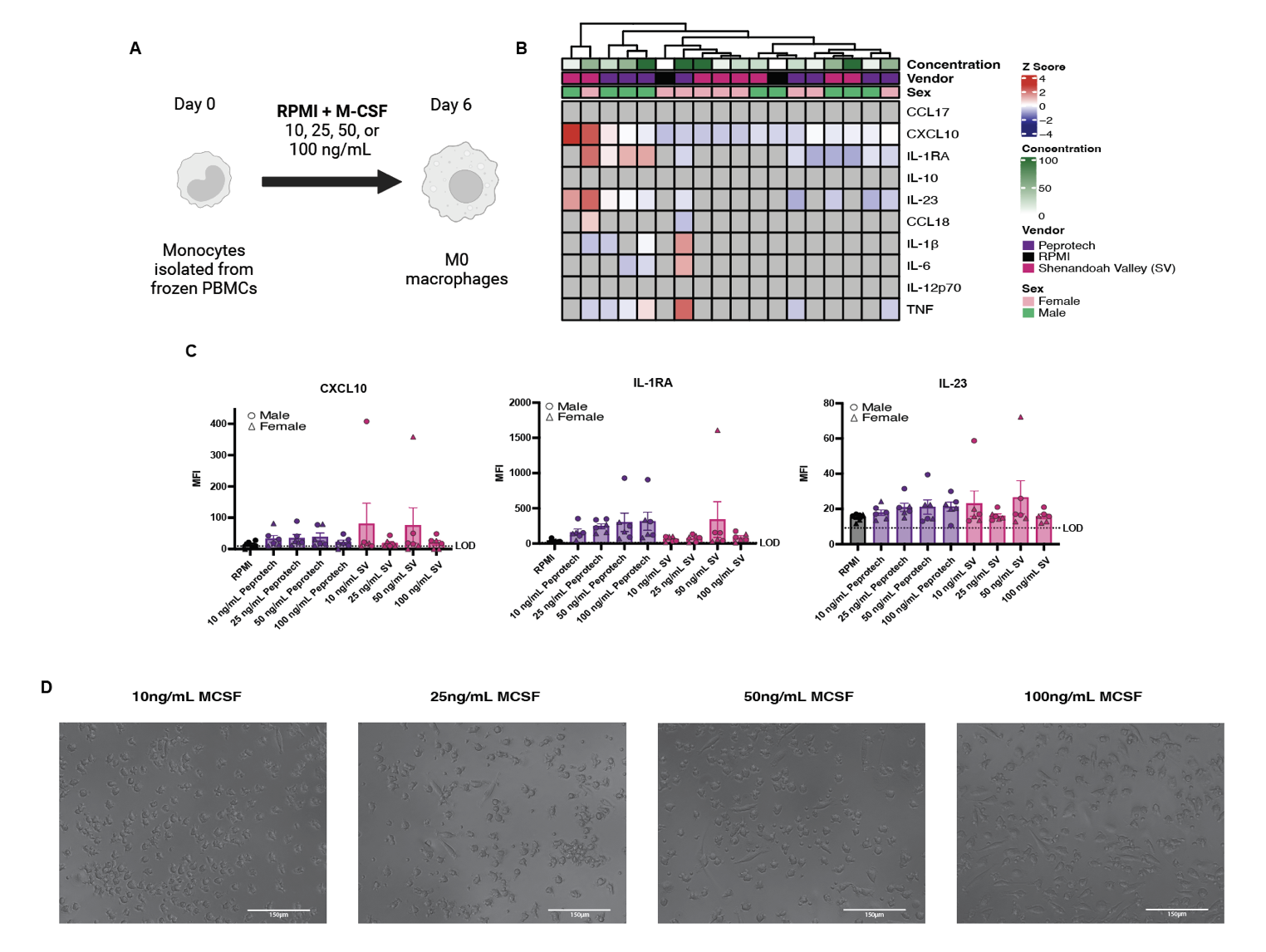


**Supplemental Figure 1: M-CSF Differentiation yields functionally active macrophages** (A) Schematic of monocyte to macrophage differentiation. (B and C) Cytokines quantified in supernatants of differentiated M0 macrophages. Limit of (LOD) is 2 standard deviations above the average of the blank readings per cytokine. (B) Heatmap shows averaged mean fluorescence intensity (MFI) of 6 donors per condition (3 per sex) that is above the limit of detection (LOD). Data beneath the LOD is represented as a grey tile. Data is row normalized, and Z scored. (C) Bar plots of measured cytokines are shown. Male and female donors are indicated by circles or triangles, respectively. Bar is at mean with SEM plotted. (D) Representative brightfield microscopy images of cultured macrophages at 120 hours.


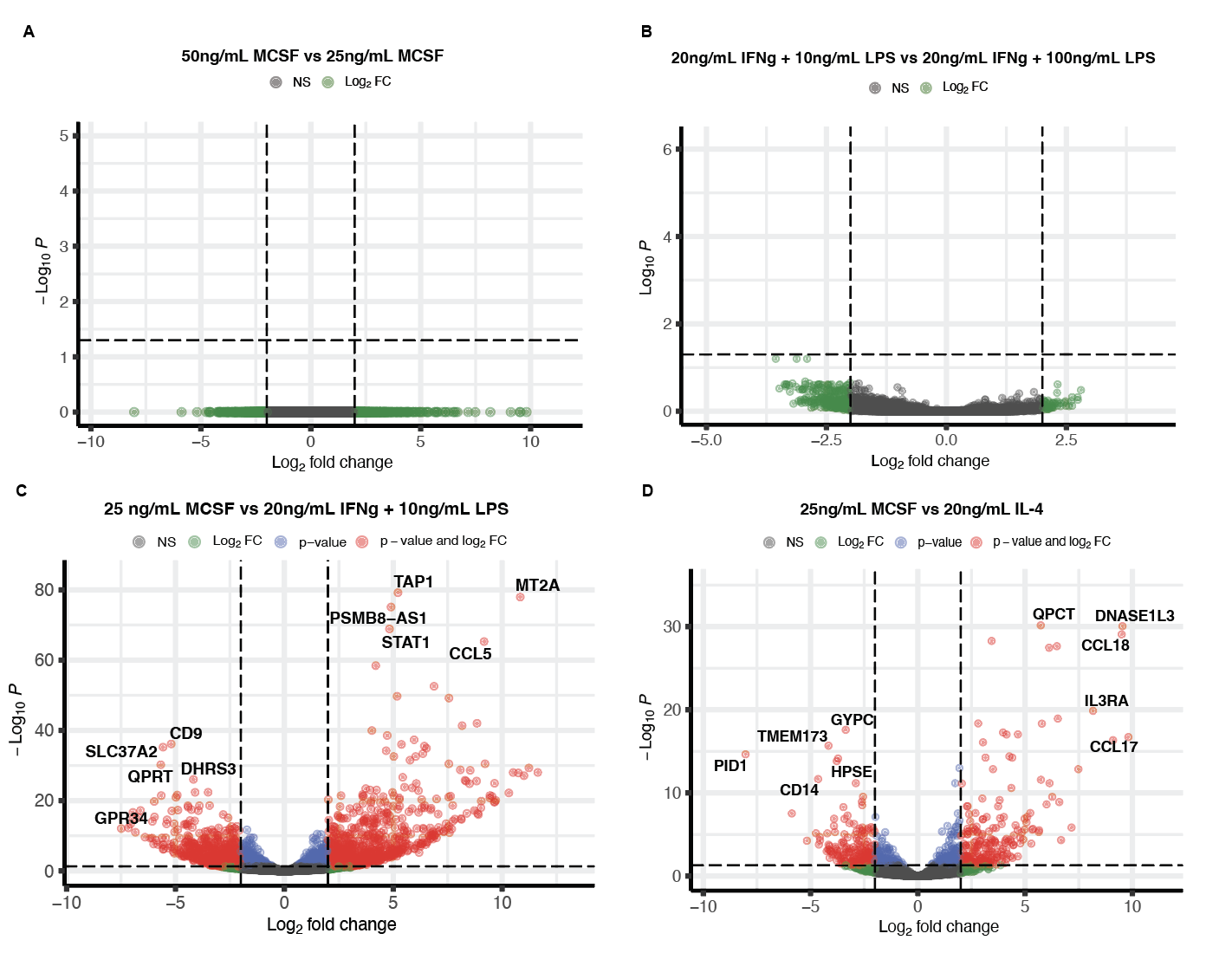


**Supplemental Figure 2: Transcriptional variation of activating stimuli with varied concentrations** (A-D) Volcano plots of differentially expressed genes of macrophages treated with variant concentrations of (n=4). (A) M0 macrophages treated with 25ng/mL or 50 ng/mL of M-CSF 2 female and 2 male donors. (B) M1-like macrophages treated with 20ng/mL IFNγ and 10 ng/mL LPS or 20 ng/mL IFNγ and 100 ng/mL LPS. (C) M1-like macrophages treated with 20 ng/mL IFNγ and 10 ng/mL LPS vs 25 ng/mL M-CSF. (D) M2-like macrophages treated with 20 ng/mL IL-4 vs 25 ng/mL M-CSF.


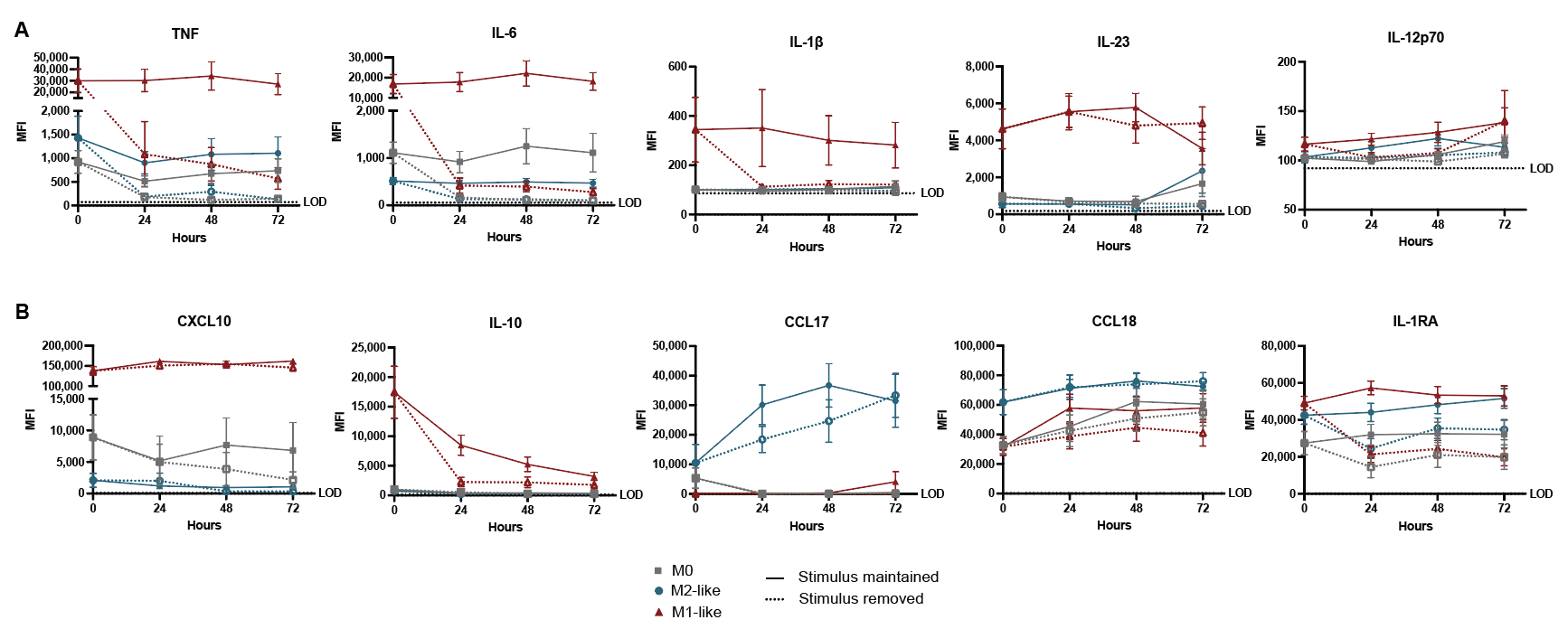


**Supplemental Figure 3: Depolarization of cultured macrophages.** Cytokine secretion of profiles of activated macrophages in the presence or absence of activating stimuli from 0 to 72 hours (n=8).


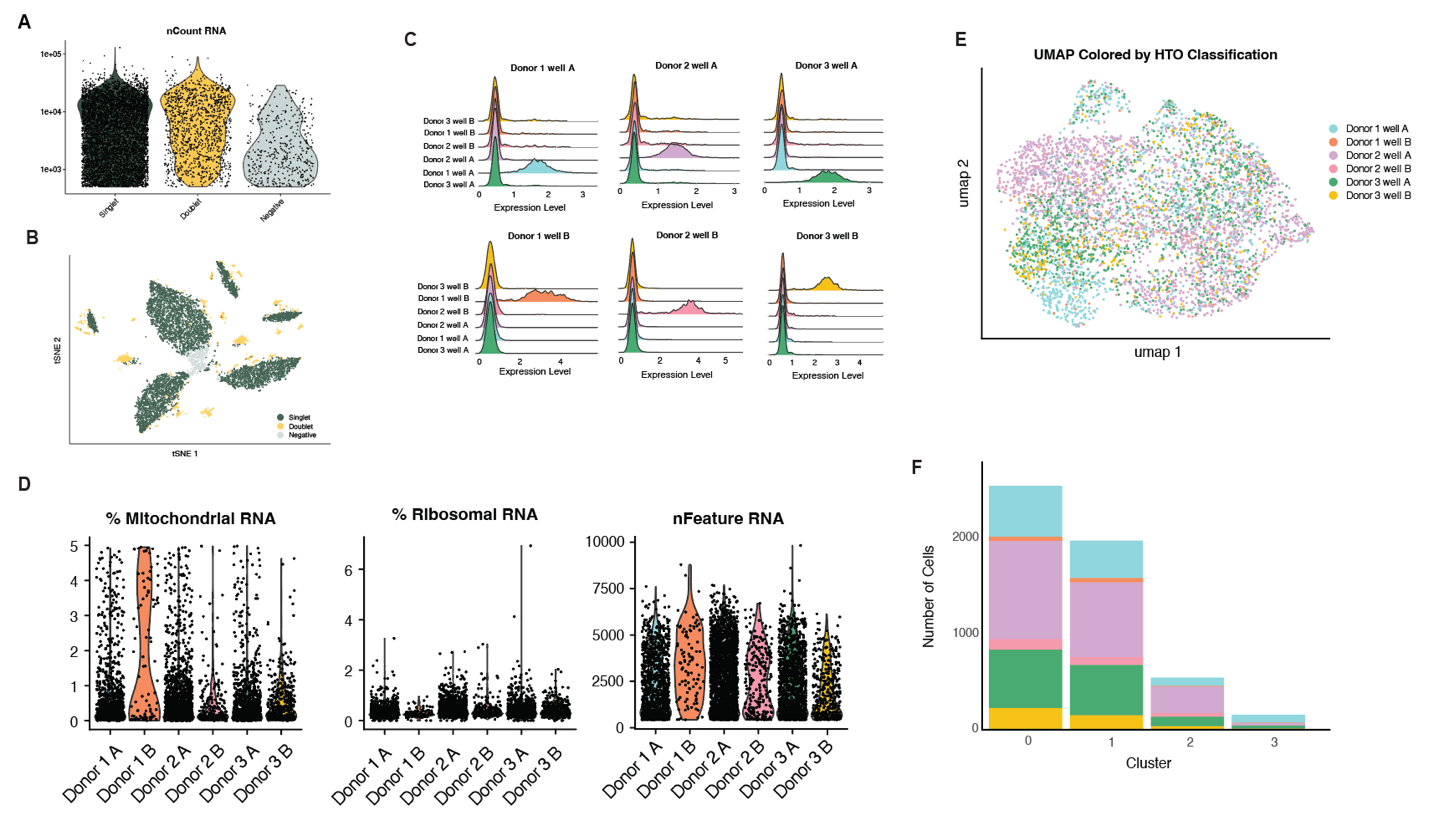


**Supplemental Figure 4: Quality statistics of snRNA-seq. (**A) Violin plots of hashtag oligo detection per cell. (B) tSNE of HTOs. (C) Ridge plots of hashtag oligo demultiplexing for all 6 wells. (D) Violin plots of RNA quality statistics split by HTO classification. (E) RNA umap colored by HTO donor. (F) Stacked bar plot of cell count per donor in each cluster.

**Supplemental Figure 5: Pathway results of snRNA-seq clusters.** A) Dot plot of genes plotted in figure 2A by cluster. B) Venn diagram of top 20 pathways per analysis captured in metascape. (C - F) Pathway analysis of clusters within M1-like macrophages in Metascape. (C) Cluster 0, (D) Cluster 2, (E) Cluster 3, (F) Cluster 4.

**Supplemental Figure 6: In vivo comparison of sn-RNAseq data.** (A-B) Data from Mulder et al mo-mac verse^49^. A) Label transfer of the macrophage populations onto our snRNAseq dataset. B) Stacked Bar plot of cells per cluster colored by tissue type. C. Module scoring for our Seurat clusters against Garrido-Trigo inflammatory bowel disease single cell profiling^50^.
